# Editorial: The role of maintenance therapy (immunotherapy, targeted therapy, etc) in cancer control and biologic behavior of recurrent/metastatic cervical cancer

**DOI:** 10.3389/fimmu.2023.1281775

**Published:** 2023-09-07

**Authors:** Xiaolin Li, Xianzheng Zhang, Aihua Li, Peng Xie

**Affiliations:** ^1^ Department of Radiation Oncology, Shandong Cancer Hospital and Institute, Shandong First Medical University and Shandong Academy of Medical Sciences, Jinan, Shandong, China; ^2^ Department of Oncology, Liaocheng People’s Hospital, Liaocheng, Shandong, China; ^3^ Department of Obstetrics and Gynaecology, Liaocheng People’s Hospital, Liaocheng, Shandong, China; ^4^ Shandong University, Jinan, Shandong, China

**Keywords:** cervical cancer, immune microenvironment, immunotherapy, PD-1, TCGA

In recent years, surgical treatment is still the standard treatment for patients with early cervical cancer. For patients with locally advanced cervical cancer, concurrent chemoradiotherapy (CCRT) is the conventional treatment mode. However, immunotherapy has brought new hope for patients with advanced and recurrent cervical cancer. Li et al. summarize a review of latest clinical trials of anti-PD-1 therapy in the treatment of recurrent or metastatic cervical cancer patients and its related mechanisms of anti-PD-1, providing prognostic biomarker information to guide the individual use of anti-PD-1 therapy. In the review, researchers detailed the objective response rate (ORR) and adverse events of anti-PD-1 related clinical research as a second line monotherapy for cervical cancer patients, including pembrolizumab, nivolumab, balstilimab, cemiplimab, cadonilimab. Most anti-PD-1 clinical researches had relatively high ORR (12.2%-33.3%), except one research reported 4% ORR. However, the any grade treatment-related adverse events were also high (63.3%-91.0%). In addition to monotherapy anti-PD-1, this review also summarizes the ways of anti-PD-1 therapy combined with other systemic therapies for patients with cervical cancer, including: combined chemotherapy, combined targeted therapy, combined with other immune checkpoint inhibitors, combined with concurrent chemoradiation therapy for local advanced cervical cancer patients. Subsequently, the article listed the mechanism analysis of anti-PD-1 treatment and other combined treatments that may benefit cervical cancer patients. Treatment can change the effect of tumor immunotherapy by inducing the expression of PD-1 or changing the tumor’s immune microenvironment. Xing et al. analyzed immunotherapy of cervical cancer from the point of view of scientometric analysis and clinical trials. The research incorporated 1,255 articles, reviews from 1999 to 2022 in the Web of Science Core Collection (WoSCC) and 296 trials from ClinicalTrials.gov and ICTRP. Various use of immunotherapy and/or combined with other therapies of clinical trails or articles have shown effectiveness of advanced cervical cancer.

The immune microenvironment plays an important role in cervical cancer. However, there is still a lack of systematic research on the immune infiltration environment of cervical cancer. Yao et al. combined the Cancer Genome Map (TCGA) and Gene Expression Synthesis (GEO) datasets to evaluate the immune microenvironment and obtained three different immune cell populations, two gene clusters and 119 differential genes and established an immune cell infiltration (ICI) scoring system. Three key genes, IL1B, cst7 and itga5, were identified in different cell types. The proliferation and invasion of cervical cancer cells can be regulated by these three genes. Wang et al. comprehensively analyze the tumor immune microenvironment of cervical cancer and to mine biomarkers related to immunotherapy and prognosis. The research divided TCGA data into 2 survival-related immune cell signature (ICSs) clusters and constructed an ICSs prognostic model according to eight immune-related genes (IRGs), which was prognostic for survival. Studies have found that oncogene (fkbp10) and gene (s1pr4) play opposite roles in biological behaviors such as cervical cancer staging.

Rotman et al. reported an article aimed to assess different PD-L1 detection methods (FISH, RNAish, IHC) and studied transcriptional regulation of PD-L1/PD-L2 expression by TCGA mRNAseq analysis. The research conducted that RNAish combined with interferon signaling evaluation, was a promising technique for immune checkpoint detection.

The mechanism of cervical cancer is complex, and different molecules have different effects on tumor microenvironment, invasion and metastasis. There is accumulating evidence that N6-methyladenosine (m6A) methylation can play a significant role in the early diagnosis and treatment of cancers. Zhang et al. and Ji et al. reported m6A modification patterns correlated with tumor microenvironments in cervical cancer and could be a powerful predictive signature. Li et al. evaluated the molecular mechanism of lymph node metastasis in cervical cancer. Compared with normal lymph node, the metastasis lymph nodes were characterized as increased CD8 T cells, NK cells, Treg cells, C1QA+ MRC1high macrophages and decreased naive T cells. The results provided new information of the mechanisms of cervical cancer metastasis.

Radiotherapy is still one of the important palliative treatments for patients with locally advanced cervical cancer or metastatic cervical cancer. Abscopal effect is an interesting phenomenon in radiobiology that causes activation of immune system against cancer cells. Traditionally, this phenomenon was known as a suppressor of non-irradiated tumors or metastasis. Ollivier et al. reviewed the potential abscopal effect of immune-radiation therapy in metastatic cervical cancer. However, the review indicated that there was no obvious proof of abscopal effect observed in advanced, recurrent or metastatic cervical cancer with the combination of RT and ICI. Therefore, prospective clinical trials are needed to further verify the results of abscopal effect. Further esperimental data is needed to explore the influence of radiotherapy fractionated dose mode on abscopal effect in cervical cancer patients, in order to maximize the effect of radiotherapy combined with immunotherapy.

## Author contributions

XL: Conceptualization, Data curation, Methodology, Writing – original draft. XZ: Investigation, Supervision, Writing – review & editing. AL: Formal Analysis, Supervision, Validation, Writing – review & editing. PX: Conceptualization, Funding acquisition, Methodology, Writing – original draft, Writing – review & editing.

